# ROS-Induced Oxidative Damage and Mitochondrial Dysfunction Mediated by Inhibition of SIRT3 in Cultured Cochlear Cells

**DOI:** 10.1155/2022/5567174

**Published:** 2022-01-19

**Authors:** Lingjun Zhang, Zhengde Du, Lu He, Wenqi Liang, Ke Liu, Shusheng Gong

**Affiliations:** Department of Otorhinolaryngology, Beijing Friendship Hospital, Capital Medical University, 95 Yongan Road, Xicheng District, Beijing 100050, China

## Abstract

Sensorineural hearing loss (SNHL) is one of the most common causes of disability worldwide. Previous evidence suggests that reactive oxygen species (ROS) may play an important role in the occurrence and development of SNHL, while its mechanism remains unclear. We cultured dissected organs of Corti in medium containing different concentrations (0, 0.25, 0.5, 0.75, 1, and 1.25 mM) of hydrogen peroxide (H_2_O_2_) and established a four-concentration model of 0, 0.5, 0.75, and 1 mM to study different degrees of damage. We examined ROS-induced mitochondrial damage and the role of sirtuin 3 (SIRT3). Our results revealed that the number of ribbon synapses and hair cells appeared significantly concentration-dependent decrease with exposure to H_2_O_2_. Outer hair cells (OHCs) and inner hair cells (IHCs) began to be lost, and activation of apoptosis of hair cells (HCs) was observed at 0.75 mM and 1 mM H_2_O_2_, respectively. In contrast with the control group, the accumulation of ROS was significantly higher, and the mitochondrial membrane potential (MMP) was lower in the H_2_O_2_-treated groups. Furthermore, the expression of SIRT3, FOXO3A, and SOD2 proteins declined, except for an initial elevation of SIRT3 between 0 and 0.75 mM H_2_O_2_. Administration of the selective SIRT3 inhibitor 3-(1H-1,2,3-triazol-4-yl) pyridine resulted in increased damage to the cochlea, including loss of ribbon synapses and hair cells, apoptosis of hair cells, more production of ROS, and reduced mitochondrial membrane potential. Thoroughly, our results highlight that ROS-induced mitochondrial oxidative damage drives hair cell degeneration and apoptosis. Furthermore, SIRT3 is crucial for preserving mitochondrial function and protecting the cochlea from oxidative damage and may represent a possible therapeutic target for SNHL.

## 1. Introduction

As life expectancy increases, sensorineural hearing loss has become more common, affecting people's living quality [1]. There are several types of sensorineural hearing loss (SNHL), such as noised-induced hearing loss (NIHL), age-related hearing loss (ARHL), ototoxic drug-induced hearing loss (ODIHL), and inherited hearing loss. ARHL or presbycusis is a progressive decline in hearing function that is the most prevalent type of SNHL in the elderly [2–5], which is characterized by higher hearing thresholds, beginning at high frequencies and spreading toward low frequencies, accompanied by the loss of HCs and spiral ganglion neurons (SGNs) from the basal to apical turn. Noise-induced hearing loss (NIHL) is the second most prevalent type of SNHL, behind presbycusis [3]. It is typically characterized by elevation in hearing thresholds and speech perception, tinnitus, and auditory processing disorders [6] due to damage to and/or death of cochlear hair cells, as well as primary auditory neurons after exposure to strong noise stimulation [7]. Platinum-based anticancer drugs and aminoglycoside antibiotics can lead to hearing loss at high frequencies and preferential damage to OHCs at the cochlea basal turn [8–10].

Reactive oxygen species (ROS) are mainly generated by the mitochondria in most mammalian cells [11, 12], including hydroxyl radicals and hydrogen peroxide (H_2_O_2_). Growing evidence has convincingly argued that ROS and oxidative stress are responsible for the pathogenesis of various cochlear disorders, especially SNHL, including noise exposure, senility, and ototoxicity [13, 14]. An immediate increase in ROS in the cochlea after noise exposure indicates that ROS are associated with early damage to cochlear hair cells and persist for 7-10 days [15]. In a model of the senescence-accelerated mouse prone 8 (SAMP8), oxidative stress and impairment in activities of antioxidant enzyme were shown to be involved in premature ARHL [16]. Moreover, ototoxic drugs can induce generation of ROS [17, 18]. On the other hand, recent studies have suggested that before overt hearing loss happened, cochlear synaptopathy, which synapses between IHCs and cochlear afferent nerve fibers are disrupted, is more common taking place [19], although there is no direct evidence that ROS could cause loss of synapses. Rodent studies have indicated excessive release of glutamate from IHCs and insufficient energy supply can lead to cochlear synaptopathy [20, 21], while this all could be induced by ROS. In this study, we hoped to provide some direct evidence between ROS and cochlear synaptopathy. Much evidence implicates that mitochondrial dysfunction contributes to the occurrence and evolution of SNHL [22, 23]. As the major inducer of ROS, it is assumed that exposure to noise, aging, and ototoxic drugs causes mitochondrial damage and, in turn, increases ROS accumulation, which eventually results in apoptosis, necrosis, and tissue damage [6]. Mitochondrial damage, lipid peroxidation, and immune inflammatory reactions are strongly associated with ROS [24–26]. ROS can also lower mitochondrial membrane potential and induce calcium overload, further damaging the mitochondria and contributing to hair cell apoptosis or necrosis [25, 27].

Given the important role ROS play in SNHL, the development and application of antioxidant drugs are becoming increasingly common. Sirtuin proteins are histone deacetylases dependent on nicotinamide adenine dinucleotide (NAD^+^); they have been shown to regulate the morphology and function of mitochondria and have become the focus of research in recent years. The NAD^+^-dependent mitochondrial sirtuin, SIRT3, has been shown to be able to deacetylate mitochondrial respiratory chain complex subunit proteins and promote energy production [28]. SIRT3 not only increases the activity of intracellular antioxidant enzymes by activating FOXO3A but also directly deacetylates MnSOD to increase the antioxidant capacity of cells and reduce intracellular ROS levels [29, 30]. A recent study has suggested that administration of NAD^+^ precursor, nicotinamide riboside, can prevent spiral ganglia neurite degeneration and NIHL, which is mediated by SIRT3 [31]. In vitro, overexpression of SIRT3 can ameliorate autophagic cell death, which is dependent on superoxide anions generated from mitochondria induced by cadmium [32]. However, the direct effect of SIRT3 in cochlear basilar membranes (CBMs) has not been documented yet.

Here, we explored the hypothesis that ROS induced by exposure to different concentrations of H_2_O_2_ may cause mitochondrial damage and loss of ribbon synapses in vitro, which may contribute to SNHL at the cellular level. In addition, we also explored the role of SIRT3 in cochlear oxidative damage in cultured organs of Corti in vitro, and possible protective mechanisms by SIRT3 involvement were investigated.

## 2. Materials and Methods

### 2.1. Animals

We purchased postnatal day 3 C57BL/6 mice from the Experimental Animal Department of the Capital Medical University. The experimental protocols conformed to the National Institutes of Health guidelines, which were revised in 1996 (NIH Publications). Our research had got permission from the Capital Medical University Animal Ethics Committee.

### 2.2. Cultures

After the skin was cleaned with 75% alcohol, the postnatal day 3 C57BL/6 mice were euthanized by cold CO_2_ inhalation and decapitated painlessly. The skin was peeled off the scalp, and the skull was cut into two pieces posterior to the eye. The cochleae were removed and placed in Leibovitz's L-15 (Procell, PM151012, Wuhan, China). The organ of Corti was separated away from the stria vascularis and then adhered to separate culture plates. Each plate contained 1 ml/well of culture medium, which comprised Dulbecco's modified Eagle's medium/Ham's F-12 (DMEM/F-12; 1 : 1 Mix) (Sigma-Aldrich, D8437, St. Louis, MO, USA) with 10% bovine serum albumin (Sigma-Aldrich, A1595, St. Louis, MO, USA) and 30 U/mL penicillin G (Sigma-Aldrich, P3032, St. Louis, MO, USA). The organ of Corti explants was cultured in a humidified atmosphere with 5% CO_2_ at 37°C.

### 2.3. H_2_O_2_-Induced Oxidative Stress

According to various studies, we chose to use H_2_O_2_ to induce oxidative stress in vitro. First, cochlear specimens were placed in a humidified incubator for 24 h. Then, the culture medium was discarded and substituted by fresh medium (DMEM/F-12) containing different concentrations of H_2_O_2_ (0, 0.25, 0.5, 0.75, 1, and 1.25 mM) for 24 h. Afterward, the medium was discarded, and the specimens were left and prepared according to subsequent studies' protocols.

Next, we evaluated the effect of SIRT3 on oxidative stress induced by H_2_O_2_ using 3-(1H-1,2,3-triazol-4-yl) pyridine (3-TYP) (MCE, HY-108331, Monmouth Junction, NJ, USA), an inhibitor of SIRT3. The organs of Corti were pretreated with 3-TYP (50 *μ*M) for 2 h prior to H_2_O_2_ treatment. A 100 mM stock solution was prepared with 3-TYP dissolved in DMSO (Beyotime Biotechnology, ST038, Haimen, China) and was kept at -80°C until use. We further diluted the stock solution with culture medium to appropriate concentration for administration. The highest concentration of DMSO should be under 0.1%. Additionally, it has been previously shown that 50 *μ*M 3-TYP does not significantly affect cell viability [32].

### 2.4. Immunofluorescence

The specimens were fixed with 4% paraformaldehyde for 2 min and then washed with PBS. Subsequently, they were incubated in 0.3% Triton X-100 containing 10% goat serum (ZSGB-BIO, ZLI-9021, Beijing, China) at room temperature for 15 min. Then, they were immunolabeled with monoclonal mouse anti-carboxyl-terminal binding protein 2 (CtBP2) (1 : 300, Abcam, ab204663, Cambridge, MA, USA) and rabbit anti-myosin VIIa antibody (1 : 300, Proteus Biosciences, 25-6790, Ramona, CA, USA) to label ribbon synapses and hair cells, respectively, and incubated overnight at 4°C. The next day, the specimens were counterstained with secondary antibodies conjugated with Alexa FluorTM/® 568 and 647 (1 : 300, Invitrogen, A21124, A21245, Molecular Probes, Carlsbad, CA, USA) for 2 h. The specimens were washed in PBS for 10 min and mounted on glass slides with 4′,6-diamidino-2-phenylindole (DAPI) (ZSGB-BIO, ZLI-9557, Beijing, China). Finally, the specimens were observed under a laser confocal microscope (Leica Microsystems, Wetzlar, Germany).

### 2.5. Hair Cells and Ribbon Synapse Counting

We quantified the outer hair cells (OHCs), inner hair cells (IHCs), and ribbon synapses with a ×63 magnification oil-immersion objective mounted on a Leica TCS SP8 laser confocal microscope (Leica Microsystems, Wetzlar, Germany). Approximately 14–15 IHCs and 42–45 OHCs were included in each visual field. Five specimens were selected from each group to measure the average, and percentages were used to describe the survival of hair cells. We obtained the mean of ribbon synapses for each IHC using the sum of CtBP2-stained puncta divided by the sum of IHC nuclei.

### 2.6. Determination of Mitochondria-Derived ROS

To evaluate ROS, explants were cultured in a medium containing MitoSOX (1 : 1000, Invitrogen, M36008, Carlsbad, CA, USA) for 30 min at 37°C. Then, they were washed gently with PBS for 10 min and mounted on glass slides with DAPI. All steps of the procedure were protected from light. The fluorescence intensity was analyzed by laser confocal microscopy with an optimal excitation wavelength of 568 nm (red) and calculated by Image J (National Institutes of Health, Bethesda, Maryland, USA).

### 2.7. Laser Confocal Microscopy

The specimens were scanned hierarchically with an interval of 0.5 *μ*m/layer by a ×63 magnification oil-immersion objective mounted on a Leica TCS SP8 laser confocal microscope (Leica Microsystems, Wetzlar, Germany). The final images were superimposed. The optimal excitation wavelengths of 568 nm (red), 647 nm (far-red), and 358 nm (blue) for DAPI were used.

### 2.8. Measurement of Mitochondrial Membrane Potential

Mitochondria extraction was performed with the Tissue Mitochondria Isolation Kit (Beyotime Biotechnology, C3606, Haimen, China) based on the manufacturer's instructions as preparation. Mitochondrial membrane potential was measured using the potential-sensitive fluorescent dye JC-1 (Beyotime Biotechnology, C2006, Haimen, China). The results were detected using a multimode plate reader (EnSpireTM, Perkin Elmer Singapore Pte. Ltd. Singapore) and expressed as the ratio of the readings.

### 2.9. Western Blot

To determine the levels of SIRT3 and the SIRT3-related proteins, FOXO3A and SOD2, western blot was adopted. We use radioimmunoprecipitation assay lysis buffer (Beyotime Biotechnology, P0013, Haimen, China) to extract total protein and an enhanced BCA Protein Assay Kit (Beyotime Biotechnology, P0010S, Haimen, China) to quantify protein concentration. Sodium dodecyl sulfate-polyacrylamide gel electrophoresis was used to isolated protein lysates. Then, we transferred protein lysates onto polyvinylidene difluoride membranes and incubated in a 5% fat-free milk blocking solution for 1 h. After washing in tris-buffered saline, the membranes were incubated with the primary antibodies anti-SIRT3 (1 : 1000 CST, D22A3, Shanghai, China), anti-FOXO3A (1 : 1000 CST, D19A7, Shanghai, China), and anti-SOD2 (1 : 1000 CST, D3X8F, Shanghai, China) at 4°C overnight. After washing for 7 min three times, the membranes were subsequently incubated with the appropriate horseradish peroxidase-conjugated secondary antibody (1 : 5000, Santa Cruz Biotechnology, SC-2357-CM, Santa Cruz, CA, USA) at room temperature for 1 h. Finally, the membranes were visualized using BeyoECL Plus (Beyotime Biotechnology, P0018S, Haimen, China). We used the Image-Pro Plus 6.0 software (Media Cybernetics, Rockville, MD, USA) to analyze quantification of these proteins.

### 2.10. Apoptosis Staining

We used a terminal deoxynucleotidyl transferase-mediated deoxy uridine triphosphate nick-end labeling staining kit TUNEL POD (Roche Molecular Biochemicals, 11772465001, Mannheim, Germany) to detect hair cell apoptosis. After being incubated in 0.3% Triton X-100 containing 10% goat serum (ZSGB-BIO, ZLI-9021, Beijing, China) at room temperature for 15 min, the specimens were immunolabeled with rabbit anti-myosin VIIa antibody (1 : 300, Proteus Biosciences, 25-6790, Ramona, CA, USA) and incubated overnight at 4°C. The next day, the specimens were first counterstained with secondary antibodies conjugated with Alexa FluorTM/® 647 (1 : 300, Invitrogen, A21124, A21245, Molecular Probes, Carlsbad, CA, USA) for 2 h. And then, the label reaction with TUNEL POD was carried out for 1 h at 37°C. Wash the specimens with PBS for 10 min and mount them on glass slides with DAPI (ZSGB-BIO, ZLI-9557, Beijing, China). Finally, the specimens were observed under a laser confocal microscope (Leica Microsystems, Wetzlar, Germany).

### 2.11. Statistical Analyses

We use GraphPad Prism 8 (GraphPad Software, Inc., La Jolla, CA, USA) to perform statistical cartography and illustrate our data with mean ± standard error. The paired-sample *t*-test was performed to evaluate differences between the different groups. Statistical significance was set at *P* < 0.05.

## 3. Results

### 3.1. H_2_O_2_ Induces Dose-Dependent Ribbon Synapse, and Hair Cells Decline: Model Building

**Figure 1:**
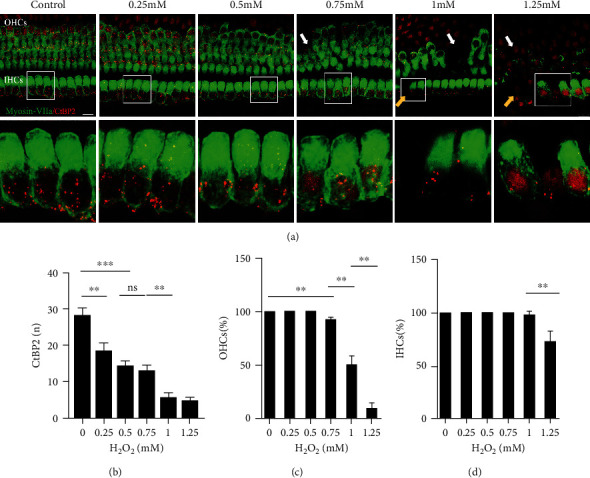
H_2_O_2_ induces dose-dependent loss of ribbon synapses and hair cells at the 0, 0.25, 0.5 0.75, 1, and 1.25 mM concentrations. (a) Confocal images showing ribbon synapses (red) and hair cells (green) in the middle region of cochlear explants at each concentration. The white arrows indicate OHC damaged areas. Yellow arrows indicate IHC damaged areas. (b) Quantification of ribbon synapses at each concentration. (c) Rate of OHC survival at each concentration. (d) Rate of IHC survival at each concentration. Data are expressed as means ± SD (*n* = 5 organ of Corti per concentration). ^∗^*P* < 0.05, ^∗∗^*P* < 0.01, and ^∗∗∗^*P* < 0.001. Scale bar = 10 *μ*m.

First, we determined the changes in the number of ribbon synapses and the survival of hair cells under different H_2_O_2_ concentrations. Our findings showed that after a 24 h exposure to H_2_O_2_, the number of ribbon synapses and cochlear hair cells was significantly decreased in a concentration-dependent manner. We recorded the concentrations at which there was apparent loss of ribbon synapses with no loss of hair cells, apparent loss of ribbon synapses and OHCs, and apparent loss of IHCs (Figure 1(a)).

The number of ribbon synapses in the middle turn of the organ of Corti in the control, 0.25, 0.5, 0.75, 1, and 1.25 mM groups was 28.2 ± 81.99, 18.5 ± 2.11, 14.36 ± 1.37, 12.96 ± 1.63, 5.62 ± 1.36, and 4.75 ± 0.92, respectively (Figure 1(b)). In contrast with the 0.25 mM group, there were significantly fewer ribbon synapses in the 0.5 mM group in comparison with the control group (*P* < 0.001). There was no significant difference in the number of ribbon synapses in the 0.5 mM and 0.75 mM groups (*P* > 0.05), while the number of ribbon synapses in the 1 mM group was efficaciously lower than that in the 0.75 mM group (*P* < 0.01). And no significant difference was found in the number of ribbon synapses between the 1 mM and 1.25 mM groups.

Regarding the survival of hair cells, we investigated the whole cochlear basilar membranes (Figures 1(c) and 1(d)). In the 0.25 mM and 0.5 mM groups, hair cells remained intact, and there were no obvious alterations in their quantities, although the arrangement of hair cells in the apex of the cochlea was disordered in the 0.5 mM group. At a concentration of 0.75 mM H_2_O_2_, 92.75 ± 1.53% of OHCs (*P* < 0.01) and all IHCs remained. In the 1 mM group, approximately 50% of OHCs survived (50.10 ± 7.99%), which was significantly different compared with the 0.75 mM group (*P* < 0.01), and 97.92 ± 2.95% of IHC survived. There was extensive hair cell loss in the 1.25 mM group; only 9.13 ± 5.54% and 72.35 ± 9.79% of OHCs and IHCs survived, respectively, which was significantly different from the 1 mM group (both *P* < 0.01). According to the results above, we built a model of four concentrations: 0, 0.5, 0.75, and 1 mM for the following study.

### 3.2. Pharmacological Suppression of SIRT3 and Aggravated Oxidative Damage to the Cochlea

To investigate the impact of SIRT3 mitigation on H_2_O_2_-induced oxidative damage, we used a selective SIRT3 inhibitor, 3-TYP [33]. Previous research by Pi et al. revealed that 3-TYP can inhibit SIRT3 activity but does not downregulate protein expression [32].

**Figure 2:**
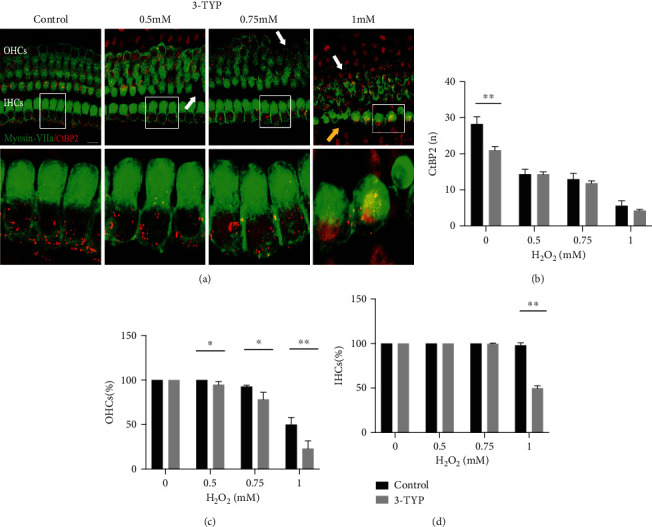
H_2_O_2_ induces aggravated oxidative damage to cochlear by suppression of SIRT3. (a) Representative images of ribbon synapses (red) and HCs (green) in the middle region of cochlear explants in 0, 0.5, 0.75, and 1 mM groups pretreated with 3-TYP. The white arrows indicate OHC damaged areas. Yellow arrows indicate IHC damaged areas. (b) Comparison of ribbon synapse at each concentration between matched and 3-TYP groups. (c) Comparison of OHC survival at each concentration between matched and 3-TYP groups. (d) Comparison of IHC survival at each concentration between matched and 3-TYP groups. Data are shown as means ± SD (*n* = 5 in each group). ^∗^*P* < 0.05, ^∗∗^*P* < 0.01, and ^∗∗∗^*P* < 0.001. Scale bar = 10 *μ*m.

We first quantified the number of ribbon synapses and analyzed the survival of hair cells in the groups established above. Our results revealed that the damage to ribbon synapses and hair cells was dependent on the concentration of H_2_O_2_ (Figure 2). In the presence of 3-TYP, the number of ribbon synapses in the middle turn of the organ of Corti in the control, 0.5, 0.75, and 1 mM groups declined with 21.03 ± 0.98, 14.31 ± 0.72, 11.85 ± 0.62, and 4.25 ± 0.33, respectively (Figure 2(b)). Interestingly, after pretreated with 3-TYP, no significant difference was discovered in the number of ribbon synapses between these H_2_O_2_ concentrations, except in the control group, in which the number was declined (*P* < 0.01) (Figure 2(b)).

However, after pretreated with 3-TYP, HCs in H_2_O_2_ groups appeared more disorganized and morphologically abnormal, as compared Figure 1(a) with Figure 2(a). Furthermore, as shown in Figure 2(c), there was a significantly lower rate of OHC survival in the 0.5 mM group compared with the group that was not treated with 3-TYP (*P* < 0.05). A similar tendency also appeared in the 0.75 mM and 1 mM groups (*P* < 0.05 and *P* < 0.01, respectively). Regarding the survival rate of IHCs (Figure 2(d)), in the 0.75 mM group, we observed that IHC loss began, and those that remained were chaotically arranged. In the 1 mM group treated with 3-TYP, only 49.91 ± 2.47% IHCs survived, compared with 97.92 ± 2.95% in the 1 mM group without 3-TYP (*P* < 0.01). However, there was no significant difference between the rate of IHC survival in both control and 0.5 mM groups. These results revealed that 3-TYP could inhibit intracellular antioxidant activity and aggravate oxidative stress, increasing the damage to the ribbon synapse and hair cells.

### 3.3. H_2_O_2_ Induces Increased Mitochondrial ROS in CBMs, and This Is Enhanced with 3-TYP Treatment

**Figure 3:**
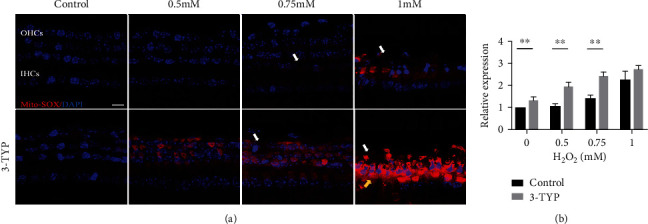
H_2_O_2_ induces ROS generation, which is enhanced by 3-TYP. (a) Confocal images of MitoSOX staining (red) in the 0, 0.5, 0.75, and 1 mM groups pretreated with or without 3-TYP. The white arrows indicate OHC damaged areas. Yellow arrows indicate IHC damaged areas. (b) Relative level of fluorescence intensity in each group. Data are expressed as means ± SD (*n* = 5 organ of Corti per concentration). ^∗^*P* < 0.05, ^∗∗^*P* < 0.01, and ^∗∗∗^*P* < 0.001. Scale bar = 10 *μ*m.

We measured the levels of mitochondrial ROS using MitoSOX staining. The red fluorescence intensity of MitoSOX staining in the groups treated with H_2_O_2_ was significantly more apparent than that in the control group, and the level was largely dependent on the concentrations of H_2_O_2_, with the highest in the 1 mM group, regardless of pretreatment with 3-TYP, as shown in Figure 3. The quantitative comparison was performed using red fluorescence intensity (Figure 3(b)). Relative staining in the 0.5, 0.75, and 1 mM groups rose by 1.07 ± 0.10 − fold, 1.42 ± 0.15 − fold, and 2.27 ± 0.37 − fold, respectively. No significant difference was shown in the relative ROS levels between the 0 and 0.5 mM groups. Relative fluorescence intensity in the 0.5 mM group was significantly lower than that in the 0.75 mM group (*P* < 0.05), and relative fluorescence intensity in the 0.75 mM group was significantly lower than that in the 1 mM group (*P* < 0.05).

After pre-treated with 3-TYP, the relative fluorescence intensity was increased in each concentration group (0, 0.5, 0.75, and 1 mM), by 1.33 ± 0.15 − fold, 1.96 ± 0.19 − fold, 2.43 ± 0.18 − fold, and 2.74 ± 0.16, respectively. Relative staining was significantly higher as H_2_O_2_ concentrations higher: control vs. 0.5 mM, *P* < 0.01, 0.5 vs. 0.75 mM, *P* < 0.05, and 0.75 vs. 1 mM, *P* < 0.05, respectively. Furthermore, the levels of relative fluorescence intensity in control, 0.5, and 0.75 mM 3-TYP pretreated groups were significantly higher than in the nontreated matched group with *P* < 0.01. Thus, it indicated that mitochondrial ROS levels increased in response to H_2_O_2_ treatment in vitro. Furthermore, this may be enhanced by inhibition of SIRT3.

### 3.4. H_2_O_2_ Induces Mitochondrial Dysfunction in CBMs, Which Is Intensified by 3-TYP Treatment

**Figure 4:**
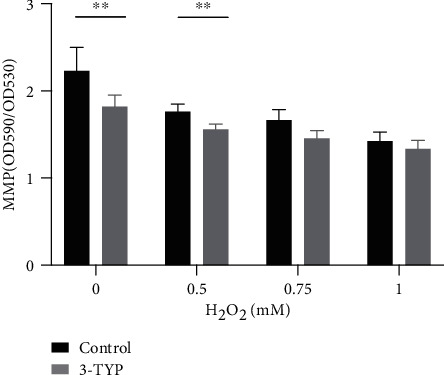
Comparison of MMP levels in the 0, 0.5, 0.75, and 1 mM groups between matched nontreated and 3-TYP-treated groups. Data are expressed as means ± SD (*n* = 5 organ of Corti per concentration). ^∗^*P* < 0.05, ^∗∗^*P* < 0.01, and ^∗∗∗^*P* < 0.001.

The MMP was used to evaluate mitochondrial function. As shown in Figure 4, MMP levels in the 0, 0.5, 0.75, and 1 mM groups were 2.23 ± 0.27, 1.76 ± 0.09, 1.67 ± 0.12, and 1.43 ± 0.10, respectively. Our results showed that mitochondrial function was inversely related to H_2_O_2_ concentration. The MMP level in the 0.5 mM and 0.75 mM groups was not significantly different. The MMP level in the 0.5 mM group was significantly lower than that in the control group (*P* < 0.01), as was the MMP level in the 1 mM group compared to that in the 0.75 mM group (*P* < 0.01). The result also illustrated the MMP levels in the same concentration groups pretreated with 3-TYP; these were 1.82 ± 0.13, 1.56 ± 0.06, 1.46 ± 0.08, and 1.34 ± 0.10, respectively, which also demonstrated a concentration-dependent decline. There was a significant difference in MMP between the control and 0.5 mM groups (*P* < 0.01) and the 0.5 mM and 1 mM groups (*P* < 0.05). To investigate the effect of SIRT3 suppression, we compared the MMP level between the 3-TYP pretreated group and nontreated group. The MMP level in each 3-TYP pretreated group was lower than in the nontreated matched group, with a significant difference in the 0 and 0.5 mM groups (*P* < 0.01). Our findings indicated that H_2_O_2_ resulted in a concentration-dependent decrease in mitochondrial membrane potential, and SIRT3 played an important role in protecting mitochondrial function.

### 3.5. H_2_O_2_ Downregulates SIRT3 and SIRT3-Dependent Protein Expression

**Figure 5:**
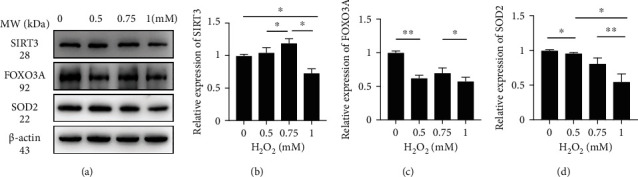
H_2_O_2_ induces downregulation of SIRT3, FOXO3A, and SOD2. (a) Representative western blot analysis using antibodies against SIRT3, FOXO3A, SOD2, and *β*-actin in CBMs. (b) Protein expression of SIRT3 in the 0, 0.5, 0.75, and 1 mM groups. (c) Protein expression of FOXO3A in the 0, 0.5, 0.75, and 1 mM groups. (d) Protein expression of SOD2 in the 0, 0.5, 0.75, and 1 mM groups. Data are expressed as means ± SD (*n* = 5 organ of Corti per concentration). ^∗^*P* < 0.05, ^∗∗^*P* < 0.01, and ^∗∗∗^*P* < 0.001.

As previously mentioned, SIRT3 is crucial in oxidation resistance and protects cells from oxidative damage. We analyzed the expression of SIRT3 and the related proteins FOXO3A and SOD2 by western blotting to confirm this effect (Figure 5). As shown in Figure 5(b), the level of SIRT3 protein was significantly higher in the 0.75 mM group than in the 0.5 mM and control groups (1.19 ± 0.07 − fold vs. 1.05 ± 0.08 − fold, *P* < 0.05; 1.19 ± 0.07 − fold vs. 1.00 ± 0.02 − fold, *P* < 0.05, respectively). However, in the 1 mM group, the protein level was dramatically decreased to 0.73 ± 0.07 − fold, which was significantly lower than in any other group (*P* < 0.05). This suggested that stimulation by H_2_O_2_ initially elevated the level of SIRT3 protein, which could help cells to resist oxidative damage to a certain degree. However, this elevation was limited and finally declined. The level of FOXO3A protein, a downstream target of SIRT3, generally declined (Figure 5(c)). By contrast with the control group, the level of FOXO3A was decreased in the 0.5 mM, 0.75 mM, and 1 mM groups by 0.62 ± 0.04 − fold (*P* < 0.01), 0.70 ± 0.07 − fold (*P* < 0.05), and 0.58 ± 0.06 − fold (*P* < 0.01), respectively. Compared with the control group, the expression of SOD2 protein declined stably to 0.96 ± 0.01 − fold in the 0.5 mM group (*P* < 0.05), 0.81 ± 0.08 − fold in the 0.75 mM group (*P* < 0.05), and 0.55 ± 0.11 − fold in the 1 mM group (*P* < 0.05). Moreover, the levels were significantly different between the 0.5 mM and 1 mM groups and the 0.75 mM and 1 mM groups, although no difference was noticed in expression levels between the 0.5 mM and 0.75 mM groups, which may be related to statistical error. The decrease in the expression of these two downstream proteins indicates that despite elevated SIRT3 expression, H_2_O_2_ activated the oxidative reaction and degraded the expression of antioxidant proteins, causing damage to tissues. Together, these results provide evidence that H_2_O_2_ could induce the downregulation of SIRT3 and SIRT3-related proteins, thus inhibiting the antioxidant reaction and causing damage.

### 3.6. Hair Cell Apoptosis Induced by H_2_O_2_ and Intensified by 3-TYP Treatment

**Figure 6:**
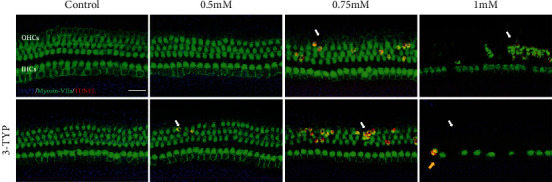
H_2_O_2_ induce occurrence of hair cell apoptosis, and this is intensified by 3-TYP. Representative images of TUNEL staining (red) and hair cells (green) in 0, 0.5, 0.75, and 1 mM groups pretreated with or without 3-TYP. The white arrows indicate TUNEL-positive cells in OHC areas. Yellow arrows indicate TUNEL-positive cells in IHC areas. Scale bar = 25 *μ*m.

As mentioned above, the loss of hair cells appeared significantly H_2_O_2_ concentration-dependent, which may due to the activation of apoptosis induced by H_2_O_2_. To confirm the occurrence of apoptosis, we used TUNEL POD staining. As shown in Figure 6, the TUNEL-positive cells were first observed in the OHC area in the 0.75 mM group. In the 1 mM group, a large number of OHCs appeared apoptosis and a portion of IHCs lost directly. After treatment with 3-TYP, apoptosis of hair cells occurred earlier and severer, as the TUNEL-positive cells were first observed in the OHC area in the 0.5 mM group and more TUNEL-positive OHCs were in the 0.75 mM group. Furthermore, in the 1 mM group, none of OHCs survived and almost half of IHCs lost directly. However, we still could observe a TUNEL-positive cell in IHC area. This suggests that H_2_O_2_ induces apoptosis of hair cells in the CBMs, and administration of 3-TYP could exacerbate the occurrence of apoptosis.

## 4. Discussion

Hair cells mainly function in turning the sound wave energy into electric signals [34]. Although SNHL could be caused by many factors, including genetic factors, aging, chronic cochlear infections, infectious diseases, ototoxic drugs, and noise exposure, most of the SNHL occurs due to the irreversible loss of HCs and spiral ganglion neurons (SGNs), both of which have limited regeneration ability in adult mammals [35]. Thus, how to protect the cochlear HC is a key scientific question in the hearing research field. As the human cochlear is inaccessible, in vitro animal models could be a crucial way to study SNHL. Here, we studied the oxidative damage to the organ of Corti caused by H_2_O_2_ and further investigated the changes of the oxidative damage after inhibition of SIRT3. Accumulation of ROS could result in various degrees of loss of hair cells and ribbon synapses, decrease of mitochondrial membrane potential, and decrease of SIRT3 and related protein expression. Furthermore, under the administration of 3-TYP, oxidative damage was aggravated.

### 4.1. Apoptosis May Contribute to Loss of HCs

It has been proven that hearing mostly depends on hair cells to convert mechanical stimulation to electrochemical activity and the IHC ribbon synapses to transmit electrochemical signals [36, 37]. OHCs are mainly responsible for the active mechanical amplification process, which facilitates high sensitivity and good frequency resolution. IHCs are responsible for sound detection and provide afferent auditory neurotransmission to the brain [11, 38, 39]. In this study, cochlear sensory hair cells and IHC ribbon synapses in cochlear explants exhibited dose-dependent cytotoxic effects when exposed to H_2_O_2_. At certain concentration group, we viewed an enormous loss of OHCs and a slight loss of IHCs. Previous research has shown that the degeneration and loss of hair cells are largely due to the activation of apoptosis [40, 41]. Apoptosis occurs in two ways: the extrinsic pathway, activated by death receptors, and the intrinsic pathway, initiated by a change in mitochondrial membrane permeability [42–44]. Mitochondrial dysfunction causes the permeability transition pore to open in an irreparable way, which subsequently leads to rise of the permeability of mitochondrial outer membrane [42, 45]. Moreover, the accumulation of ROS, decrease of the mitochondrial membrane potential, and release of apoptosis-related factors and cytochrome c were induced by mitochondrial dysfunction spark apoptotic and necrotic cell death pathways as well [44, 46].

### 4.2. Lack of Energy May Contribute to Synaptic Loss

Our results indicate that the intrinsic pathway of apoptosis due to mitochondrial dysfunction and overgeneration of ROS may contribute to the loss of hair cells in the cochlea. Our results also showed that exposure to H_2_O_2_ significantly decreased the number of ribbon synapses, even at low concentrations. The concomitant decline in the mitochondrial membrane potential decreases adenosine triphosphate (ATP) levels in the cochlea, which becomes insufficient for the transportation of ribbon synaptic vesicles and maintenance of ribbon synapses' function [41]. Considering oxidative stress, related studies have shown that inhibition of AMPK, a key cellular energy sensor related to ATP production, could attenuate the loss of outer hair cells and ribbon synapses and preserved auditory function after noise exposure [47]. Recent animal studies have shown that synaptic loss can occur without permanent hearing threshold shifts after noise exposure [48, 49], suggesting that synaptopathy is a significant marker of early noise-induced hearing loss.

### 4.3. Accumulation of ROS Reduces MMP and Triggers Oxidative Damage

To evaluate the level of ROS, we used MitoSOX staining, and our results demonstrated that the higher the concentration, the greater the accumulation of ROS and the more severe the damage to the cochlea. Most mammalian cells generate ROS, which are regarded as toxic products of cellular metabolism by the mitochondria, and can act as signalling molecules to regulate various physiological processes [50]. It is assumed that noise exposure induces mitochondrial damage and, in turn, increases ROS accumulation. A considerable amount of ROS can trigger progressive oxidative damage, promote oxidation of the mitochondrial DNA and proteins [51], and induce lipid peroxidation products, which can result in apoptosis and reduce cochlear blood flow [6]. Normal mitochondrial membrane potential is a prerequisite for maintaining oxidative phosphorylation of mitochondria and ATP production. The cumulative burden of ROS production can lead to MMP breakdown, which further results in energy deficiency and mitochondrial dysfunction [41, 52]. In this study, the MMP levels in both groups (with and without 3-TYP) decreased as H_2_O_2_ concentration increased. In particular, the pharmacological inhibitor 3-TYP of SIRT3 intensified the decline of MMP.

### 4.4. Potential Role of SIRT3 in Hearing Protection and Mitohormesis

SIRT3 is the major mitochondrial deacetylase and is expressed in metabolically active tissues such as the liver, kidney, and heart [28]. It appears that it can resist various mitochondrial stresses, especially by utilizing cellular antioxidant systems to combat oxidative stress. FOXO3A is a forkhead transcription factor that is deacetylated by SIRT3 to increase the transcription of key antioxidant genes, including SOD2 and catalase, and protects mitochondria from further oxidative stress [53, 54]. SOD2 is responsible for reducing ROS and protecting against oxidative stress and is also activated by SIRT3-mediated deacetylation [30]. Additionally, SIRT3 directly deacetylates mitochondrial isocitrate dehydrogenase, IDH2, and stimulates its activity [55], which helps to maintain the mitochondrial pool of NADPH for conversion of reduced glutathione [56–58].

To detect the expression of SIRT3, FOXO3A, and SOD2 proteins, we performed western blot analysis. Our results revealed that under moderate amounts of ROS (i.e., the 0.5 mM and 0.75 mM H_2_O_2_ groups), the level of SIRT3 initially increased and subsequently decreased in the 1 mM group. We suggest that this may correspond with mitohormesis, which means that the effects caused by a stressor may be beneficial when its level is relatively low and deleterious when it is high [59]. It has been reported that the level of SIRT3 increases under calorie restriction (CR), fasting, and exercise training in different tissues [55, 60]. Under calorie restriction, a moderate increase in ROS can trigger oxidative stress resistance activities to attenuate oxidative cellular damage [59, 61]. However, large or chronic increases in ROS may result in damage or cell death, as they exceed the capacity to maintaining homeostasis; thus, the balance is broken. It is noteworthy that oxidative stress is commonly associated with mitochondrial hyperacetylation [60, 62]. We assume that the activities of antioxidants and related enzymes such as FOXO3A and SOD2 are dampened through direct acetylation [58, 63, 64]. In this study, we found that the levels of FOXO3A and SOD2 decreased as the H_2_O_2_ concentration increased. As mentioned above, a moderate ROS rise increases SIRT3 transcription, thus helping to achieve a new hormetic steady state. The increased levels of SIRT3 can then deacetylate FOXO3A and SOD2 to defend against oxidative stress, but this capacity is limited. Mitochondrial hyperacetylation fails in the function of antioxidant enzymes and excess neutralization with oxidation products and ultimately reduces the expression of SIRT3, FOXO3A, and SOD2 and worsens oxidative stress. Interestingly, the expression of FOXO3A protein rose slightly in the 0.75 mM H_2_O_2_ group, which may probably due to dramatical expression of its upstream protein SIRT3, as assumed. Also, statistical bias cannot be ruled out, either.

To provide direct evidence that SIRT3 is a key factor in the preservation of mitochondrial function and antioxidation, we performed biochemical experiments with the selective SIRT3 inhibitor, 3-TYP. 3-TYP inhibits SIRT3 activity but does not affect SIRT3 protein expression [32, 33]. Our findings illustrated that inhibition of SIRT3 exacerbated the level of ROS, loss of hair cells and ribbon synapses, and decreased MMP. A previous study in cultured HepG2 cells had suggested that activation of SIRT3 could suppress mitochondrial-derived ROS-stimulated autophagic cell death induced by cadmium through SIRT3/SOD2 pathway [32]. ROS is important for osteoclasts in differentiation and activation induced by receptor activator of NF-*κ*B ligand (RANKL). Suppression of SOD2 activity by SIRT3-targeted siRNA could increase ROS levels and raise osteoclastogenesis [65]. High expression of xCT, which is the member 11 of solute carrier family 7 (SLC7A11), was commonly along with increased levels of ROS and sensitivity to glucose deprivation in breast cancer cells. Downregulation of SIRT3 further increased the levels of ROS and promoted xCT-related cell death [66]. Adjudin, a lonidamine analogue, could protect cochlear HCs from gentamicin-induced damage mediated by the SIRT3-ROS axis in vitro [67]. Furthermore, an in vivo study revealed that SIRT3^−/−^ mice were more susceptible to NIHL, and SIRT3 ^−/−^ mice treated with nicotinamide riboside exhibited less protection from both TTS and PTS after noise exposure [31]. On the other hand, it is interesting that significant reduction in the number of ribbon synapses was only discovered in the control group pretreated with 3-TYP. As mentioned above, great evidence shows that the level of SIRT3 increases under stress like CR. We have reason to believe that the protective effect for ribbon synapses of higher expression of SIRT3 induced by H_2_O_2_ is stronger than inhibition of activity, while in the control group, only the activity of SIRT3 was inhibited, and the loss of ribbon synapses became severer.

However, in vitro research provides the suggestion for studies in vivo but is different from in vivo research because of the complex internal environment and variable influencing factors. Next, we hope to further study the mitochondrial oxidative damage and deacetylation of SIRT3 in NIHL in vivo.

## 5. Conclusion

Overall, we propose that ROS and oxidative stress are major causes of SNHL. Furthermore, SIRT3 is crucial for preserving mitochondrial function and protecting the cochlea from oxidative damage. Additionally, we imitate different phases of SNHL in the organ of Corti in vitro and establish a novel in vitro model for investigation of the mechanisms of SNHL.

## Data Availability

The data that support the findings of this study are openly available from the corresponding author upon reasonable request.
